# ARS: Adaptive Robust Synchronization for Underground Coal Wireless Internet of Things

**DOI:** 10.3390/s20174981

**Published:** 2020-09-02

**Authors:** Kuiyuan Zhang, Mingzhi Pang, Yuqing Yin, Shouwan Gao, Pengpeng Chen

**Affiliations:** 1 School of Computer Science and Technology, China University of Mining and Technology, Xuzhou 221116, China; zhangkuiyuan@cumt.edu.cn (K.Z.); MingzPang@cumt.edu.cn (M.P.); yinyuqing@cumt.edu.cn (Y.Y.); gaoshouwan@cumt.edu.cn (S.G.); 2 Mine Digitization Engineering Research Center, Ministry of Education, Xuzhou 221116, China

**Keywords:** adaptive robust synchronization, kalman filtering, wireless internet of things, underground coal mines

## Abstract

Clock synchronization is still a vital and challenging task for underground coal wireless internet of things (IoT) due to the uncertainty of underground environment and unreliability of communication links. Instead of considering on-demand driven clock synchronization, this paper proposes a novel Adaptive Robust Synchronization (ARS) scheme with packets loss for mine wireless environment. A clock synchronization framework that is based on Kalman filtering is first proposed, which can adaptively adjust the sampling period of each clock and reduce the communication overhead in single-hop networks. The proposed scheme also solves the problem of outliers in data packets with time-stamps. In addition, this paper extends the ARS algorithm to multi-hop networks. Additionally, the upper and lower bounds of error covariance expectation are analyzed in the case of incomplete measurement. Extensive simulations are conducted in order to evaluate the performance. In the simulation environment, the clock accuracy of ARS algorithm is improved by 7.85% when compared with previous studies for single-hop networks. For multi-hop networks, the proposed scheme improves the accuracy by 12.56%. The results show that the proposed algorithm has high scalability, robustness, and accuracy, and can quickly adapt to different clock accuracy requirements.

## 1. Introduction

Clock synchronization is a very important and critical component in wireless Internet of things (IoT) [1]. For underground coal wireless IoT, it is not guaranteed to provide a unified global clock for each device due to the physical decentralization. The sensor nodes are required in order to maintain their local clock modules. Clock synchronization is to keep the clocks relatively consistent. In many applications, such as data collection, flow control, positioning, and node data fusion, clock synchronization plays an important role [2]. Therefore, clock synchronization is one of the key technologies in wireless IoT [3,4]. The research on clock synchronization technology in underground mines is still a challenge.

Nowadays, clock synchronization in underground mines faces the following problems: because of the low cost of sensors, the stability of clock readings that come from oscillators is limited (due to aging and observation noise, etc.) [5]. The low-cost crystal oscillators in the underground mines are prone to being affected by their working conditions, such as voltage, temperature, and humidity, and may deviate significantly from the reference sources [6]. Moreover, the requirements of clock accuracy are different in many applications. Some sensors require high synchronization accuracy to cooperate to complete information collection works. Some require low accuracy and sensor resources. Therefore, an ideal clock synchronization scheme should minimize the count of message exchanges while maintaining high synchronization accuracy [7]. However, it is difficult to ensure that the sensor nodes have on-demand clock synchronization accuracy.

On the other hand, due to factors, such as time-varying channel gain and channel interference for wireless communication links in underground mines, data packets may collide, and transmission errors may cause data packet loss during wireless network transmission, which is called unreliable IoT. The clock synchronization is completed in the unreliable IoT. During the information observation process, the clock synchronization measurement data packets containing time information cannot be successfully reached within the specified observation period, which causes the instability of clock synchronization and seriously reduces the accuracy of clock synchronization.

Previous researches are usually focused on how to perform clock synchronization to improve clock accuracy (such as Lightweight Tree-Based Synchronization(LTS), Reference Broadcast Synchronization(RBS), Timing-Sync Protocol for Sensor Networks(TPSN), etc.) [8,9]. Although clock accuracy is on the order of microseconds, they cannot balance service performance and energy efficiency, that is to say, achieve better synchronization with fewer communication costs [10,11]. Essentially, the high accuracy of the clock comes at the expense of extra energy for communication and measurement. Therefore, some studies have shown that the universal clock synchronization scheme should be on-demand synchronization (ODS) [12] in order to achieve the best cost performance.

Based on the idea of on-demand synchronization [12], an universal clock model is established based on the uncertainty of clock for wireless IoT in underground mines. Additionally, a novel adaptive robust synchronization (ABS) framework based on Kalman filter is designed to determine the sampling period to achieve the customized accuracy. The design of this paper is to analyze [13] the upper and lower bounds of error variance with the unreliable link clock by Kalman filter, and introduce the uncertainty driving mechanism to adjust the synchronous sampling period of clock adaptively. This is not the traditional periodic synchronization, but the communication overhead is minimized when the clock accuracy is reached. In addition, in order to reduce the influence on the accuracy of clock, the ARS scheme can eliminate outliers in data packets containing time-stamps [14,15,16]. In a word, the main contributions of this paper are as follows:

- To the best of our knowledge, we are the first to investigate the adaptive clock synchronization for wireless IoT in underground mines, which resolves the packet loss and possible outliers based on Kalman filtering.
- The synchronization scheme is proposed for both single-hop and multi-hop networks. Moreover, the upper and lower bounds of error covariance expectation are analyzed in the case of incomplete measurement.
- We provide multiple simulation experiments to verify the above theoretical results. Because multi-hop clock synchronization is common in real life, we can extend the proposed synchronization strategy to multi-hop network scenarios.

The rest of this paper is arranged, as follows. Section 2 shows the related work of clock synchronization technology. Section 3 introduces the basics of clock synchronization and its model. The key technologies and improvement methods are described in Section 4. Section 5 reports simulation experiments and results. Finally, Section 6 summarizes the full text.

## 2. Related Work

In previous studies, various methods have been proposed to realize clock synchronization in IoT. RBS [8] proposes a receiver-receiver model that achieves relative synchronization through broadcasting beacon packets, reduces the uncertainty of wireless communication link, and reduces the delay of sender. TPSN [9] uses a sender-receiver model to eliminate propagation delay, improve clock accuracy, and good scalability. LTS is similar to TPSN. It uses a layered tree-based sender-receiver model to reduce clock synchronization complexity, thereby further improving clock accuracy.

Tavares Bruscato et al., (2017) focused on the second synchronization problem, proposing a time synchronization service for low-power WSN using low frequency real-time clocks to make the clocks of the sensor nodes converge as quickly as possible [17]. It explores the minimum number of synchronization messages by means of a smart clock update strategy with the requirement to keep low energy consumption. Castillo-Secilla et al., (2017) focused on improving time synchronization under deployments with temperature variations [18]. The clock skew of two nodes is composed of a multiplicative combination of two main components: the cut of the crystal of each oscillator and the different temperatures affecting the nodes. A correction factor that is based on temperature is proposed by applying a nonlinear filter. Masood et al., (2017) proposed a dynamic random time synchronization scheme (DISTY) [19,20], which uses the dynamic random model to track the instability of clock and synchronize the clock between nodes. Qiu et al., (2018) proposed a root node selection algorithm called R-Sync [20,21]. It uses two timers that one is used to join the synchronization network, and the other is used for clock synchronization through two-way message exchange. R-Sync performs well in terms of energy-saving and accuracy. Xuxin Zhang et al., (2019) proposed a clock synchronization scheme based on maximum consensuses, which is called Robust Maximum Time Synchronization (RMTS) [20]. The proposed RMTS scheme adjusts the software parameters that are based on the estimation of relative skew and offset so that all nodes in the network can keep synchronization. The challenges of communication delay and packet loss can be overcome.

Previous researches on clock synchronization always focus on improving the accuracy of the clock. Instead of focusing on how to improve the accuracy of clock synchronization, Ziguo Zhong et al., (2011) proposed an on-demand clock synchronization method (ODS) [12], a design for determining when to synchronize time to achieve custom accuracy. ODS describes the method to adjust clock synchronization intervals adaptively, rather than traditional periodic synchronization, in order to meet the required accuracy while minimizing communication overhead. In addition, ODS scheme can predict the accuracy of clock. On the other hand, ACES [22] is proposed to use complex Kalman filtering and periodic sampling adaptive for clock skew tracking, but obtaining accurate parameters of Kalman filtering is a difficult challenge. Qiang Liu et al., (2010) proposed Ada-Synch [23], an adaptive clock synchronization scheme. It is a parameter estimation method using the expectation maximization (EM) algorithm based on Kalman filtering. For unreliable wireless link communication, Ting Wang et al., (2019) established a Tubes-MPC [13] clock synchronization method that is based on the predictive control method with the output feedback model, the stable quantitative convergence performance of the clock synchronization index is achieved. The model prediction optimization model is established under the set of constraints, and the quantitative convergence performance of the stability of robust packet loss synchronization index under the clock is achieved. Seo et al., (2019) proposed an improved time-synchronization algorithm [24]. The improvement of time synchronizing performance was achieved by introducing a stochastic model-based direct compensation of the disturbance effects appearing in the IEEE 1588 Precision Time Protocol (PTP)-based time synchronization system.

There is little research on clock synchronization based on underground mine. Xu et al., (2013) put forward a time synchronization algorithm of linear WSN based on the clustering structure [25]. The algorithm uses clock drift compensation and abnormal data filtering technology to reduce the network time synchronization error and balance energy loss of the entire network based on the traditional clustering algorithm. Chen et al., (2014) proposed a method of transmission delay of time synchronization information for the Internet of things based on passive measurement [26]. A class of evaluation node is added to the perception layer of coal mine underground for overhearing time synchronization packets passively among perception node. The parameters under different distribution regularities are estimated by the evaluation nodes based on the maximum likelihood estimation method. Tan et al., (2019), designed a clock synchronization scheme based on a dynamic superframe according to the hybrid topology of underground mining [27]. To improve the TPSN and RBS algorithm, the base-station and sensor have different synchronization methods, respectively, and adjusts the period of the superframe dynamically by estimating the clock offset.

## 3. Kalman Filter Based Clock Model

The complexity of clock offset has prompted us to research the clock model in deep, so that clock synchronization can be performed effectively. The frequency of an oscillator is the key of clock [23], which can directly influence the accuracy of clock. In this part, this paper delves into the clock model. Firstly, the classical clock model is shown. Subsequently, this paper introduces the universal clock model that is based on the classical clock model that the complexity of clock can be described better.

Before introducing the model, the symbols are explained first. As shown in Table 1:

### 3.1. Classical Clock Model

It is known that the clock is composed of a counter and an oscillator. The characteristics of counter and oscillator determine the performance of clock. The initial offset between two clocks is controlled by the initial value of counter. Additionally, the speed of clock is controlled by the frequency of oscillator. Because it is impossible for two oscillators to vibrate precisely at the same frequency, each clock moves at different speeds. Next, the clock is defined and modeled by considering all of these factors.

#### 3.1.1. Continuous-Time Clock Model

The ideal time *t* reported by the clock can be recorded as C(t). The time difference between the given clock and the ideal clock is called clock offset. It is defined as θ(t) and it can be expressed by:

(1) θ(t)=C(t)+t

The instantaneous change rate of clock offset is called clock skew α [22,28]. Compared with the ideal clock, it is the slope of clock offset change. The clock skew could be calculated as:

(2) $$\alpha \left(t\right)=\frac{d(\theta )}{dt}\approx \frac{\theta (t+\tau )-\theta (t)}{\tau }$$

The slope of θ(t) is corresponding to the constant skew if the clock is completely stable. While this is not the case in limited IoT. The nonlinearity and state noise of oscillator will change the clock period, causing the clock skew to change over time. In addition, the influence of aging and temperature also change the frequency of oscillator.

In this section, this paper assumes that the reference node has an ideal clock module. When synchronization node deviates from the reference node, it is easy to infer the clock offset. The clock offset is divided into three separate parts [23]: instantaneous clock skew α(t), initial clock offset $\theta_{0}$, and random state noise δ(t). Therefore, the formula of clock offset θ(t) at time *t* is:

(3) $$\theta \left(t\right)=\int_{0}^{t}\alpha \left(\tau \right)d\tau +\theta_{0}+\delta \left(t\right)$$

#### 3.1.2. Discrete-Time Clock Model

Practically, it is feasible that the continuous-time clock model becomes discrete-time model after sampling. Because clock synchronization is usually achieved by exchanging messages with timestamp. Additionally, they are only discrete samples of continuous time. According to the above formula, the discrete-time clock offset is obtained by [29,30]:

(4) $$\theta \left[n\right]=\sum_{k=1}^{n}\alpha \left[k\right]\tau \left[k\right]+\theta_{0}+\delta \left[n\right]$$

where [k] represents the sampling index and τ[k] is the *k*th sampling period.

Because δ[n] is mainly caused by state noise, this paper assumes that δ[n] is an independent Gaussian distribution with covariance $\sigma_{\delta }^{2}$, so the samples is used to estimate the variance value. Rewrite the model using recursion:

(5) θ[n]=θ[n−1]+α[n]τ[n]+ζ[n]

where ζ[n]=δ[n]−δ[n−1]. In addition, ζ[n] is zero-mean white Gaussian random variable with covariance $\sigma_{\zeta }^{2}=2\sigma_{\delta }^{2}$.

### 3.2. Practical Universal Clock Model

The skew model is studied deeply according to the characteristics of oscillator, and then establish the universal clock model, in order to describe the acceleration offset from the reference node. Clock skew is affected by two factors: phase noise and natural frequency of the oscillator. The instantaneous frequency of clock can be described as [23]:

(6) $$f\left(t\right)=\int_{0}^{t}V_{f}\left(\tau \right)d\left(\tau \right)+f_{0}+\frac{1}{2\pi f_{0}}\frac{d\phi (t)}{dt}$$

where $V_{f}$ is the frequency variance, $f_{0}$ is natural frequency, and ϕ(t) is the phase noise that will affect the frequency $\frac{1}{2\pi f_{0}}\frac{d\phi (t)}{dt}$ of oscillator.

Because the frequency of oscillator can be mapped to clock skew, the relationship of above formula is converted into describing clock skew:

(7) $$\alpha \left(t\right)=\int_{0}^{t}\beta \left(\tau \right)d\tau +\alpha_{0}+\varsigma \left(t\right)$$

where β(t) is the aging rate of clock skew, $\alpha_{0}$ is the initial value, and the skew noise ς(t) corresponds to $V_{f}\left(\tau \right)$, $f_{0}$, and $\frac{1}{2\pi f_{0}}\frac{d\phi (t)}{dt}$ respectively.

Similarly, the discrete-time model of clock skew α[n] at the *n*th sampling can be written as:

(8) $$\alpha \left[n\right]=\sum_{k=1}^{n}\beta \left[k\right]\tau \left[k\right]+\alpha_{0}+\varsigma \left[n\right]$$

where β[k] is the clock aging rate, $\alpha_{0}$ is the initial value of clock skew, and ς[n] is the skew state noise. The recursion is used to rewrite the instantaneous clock skew as:

(9) α[n]=α[n−1]+β[n]τ[n]+μ[n]

where μ[n] is Gaussian white noise with covariance $\sigma_{\mu }^{2}=2\sigma_{\varsigma }^{2}$. Note that this discrete-time clock model is universal. The aging rate is equal to zero for the relatively stable oscillators, and then the clock skew can be described as constant. On the contrary, this clock model is still feasible if the aging rate is equal to the value changing with time.

When considering the above formula, the universal model has been established for clock skew. The model similar to accelerated motion is [23]:

(10) $$\theta \left[n\right]=\theta \left[n-1\right]+\alpha \left[n\right]\tau \left[n\right]+\frac{1}{2}\beta \left[n\right]\tau^{2}\left[n\right]+\xi \left[n\right]$$

where ξ[n] is white Gaussian random variable with covariance $\sigma_{\xi }^{2}$, and it is consisted of aging noise, skew noise, and offset noise. If the aging rate is ignored, the clock model is equivalent to classical clock model.

Clock synchronization can be effectively realized by tracking offset. Kalman filtering is an effective recursive tracking method. In this section, two Kalman filters are introduced based on the proposed clock model to track time-varying offset. Additionally, then, an on-demand clock synchronization estimation method is proposed to adjust the sampling period in the Kalman filtering model.

### 3.3. Observation Model Establishment

**Figure 1:**
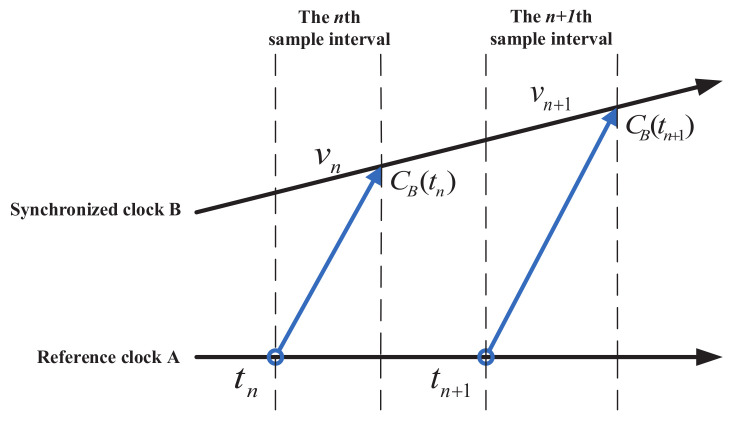
Single-hop Clock Synchronization Process.

This paper uses one-way message delivery based on sender-receiver. Figure 1 shows the clock synchronization process:

The reference node A sends a time-stamped message to the synchronous node B at local time $t_{n}$, as shown in Figure 1. Assume that node B receives the above message at local time $C_{B}\left(t_{n}\right)$. Then the clock offset formula is estimated as:

(11) $$\theta_{n}=C_{B}\left(t_{n}\right)-t_{n}$$

This paper have ignored the influence of many uncertain factors of the wireless channel, but, in practical applications, there are many uncertain random delays and random noises in the networks. Therefore, various random delays and random noise are collectively defined as measurement noise $v_{n}$.

Usually, this paper assumes that $v_{n}$ is a Gaussian random variable with independent and identical distributions, with a mean of zero and a variance of $\sigma_{v}^{2}$. The observation formula of clock offset between node A and B can be expressed as:

(12) $$z_{n}=C_{B}\left(t_{n}\right)-t_{n}+v_{n}=\theta_{n}+v_{n}$$

In this paper, $\varepsilon_{k}$ is defined to describe the packet loss event of the first round of information exchange [31,32]. When the nodes successfully exchange data, there is $\varepsilon_{k}=1$; otherwise, $\varepsilon_{k}=0$. If the information exchange process of all sampling periods is considered, $\varepsilon_{k}$ represents a random event and it conforms to the random process of Bernoulli distribution. In a statistical sense, the Bernoulli variable $\varepsilon_{k}$ has $p(\varepsilon_{k}=1)=\lambda$, where λ represents the packet arrival rate. The method is simple in thought, easy to implement, has low energy consumption and high efficiency, and can meet some wireless network applications.

### 3.4. Kalman Filtering Model of Clock

Based on the model that is proposed above, the clock model of Kalman filter [22,23] is defined, as follows:

(13) x[n]=Ax[n−1]+ω[n−1]

(14) z[n]=Hx[n]+v[n]

where x[n] is the *n*th state variable of clock; z[n] is the *n*th measurement variable; the state transition matrix is *A*; the observation matrix is *H* that maps state vector to observation vector; and, ω[n] and v[n] are Gaussian noise with covariances *Q* and *R*, respectively. In addition, two clock models is described based on the different aging rate [23].

**Situation** **1.** *When the aging rate is equal to zero, the clock model can be regarded as the classical model. According to the above formula, the Kalman filter is set to: $x\left[n\right]={\left(\begin{array}{cc} \theta [n] & \alpha [n] \end{array}\right)}^{T}$, $A=\left(\begin{array}{cc} 1 & \tau \\ 0 & 1 \end{array}\right)$, $H=\left(\begin{array}{cc} 1 & 0 \end{array}\right)$. Furthermore, $\omega \left[n\right]={\left(\begin{array}{cc} \xi [n] & \mu [n] \end{array}\right)}^{T}$ is state noise and the measurement noise is v[n], where ${\left(\cdots \right)}^{T}$ is the transpose of matrix. In terms of clock skew, this is the first-order Kalman filtering model.*

**Situation** **2.** **([33]).** *When the aging rate is constant, the clock model is regarded as the acceleration model. In this case, this paper consider that the aging rate is a random process. Assuming that the perturbation is white Gaussian noise, the Kalman filter can be set to: $x\left[n\right]={\left(\begin{array}{ccc} \theta [n] & \alpha [n] & \beta [n] \end{array}\right)}^{T}$, $A=\left(\begin{array}{ccc} 1 & \tau & \frac{1}{2}\tau^{2}\\ 0 & 1 & \tau \\ 0 & 0 & 1 \end{array}\right)$, $H=\left(\begin{array}{ccc} 1 & 0 & 0 \end{array}\right)$. Besides, $\omega \left[n\right]={\left(\begin{array}{ccc} \xi [n] & \mu [n] & \rho [n] \end{array}\right)}^{T}$ is state noise, where ρ[n] is aging rate noise and v[n] is the measurement noise. It is the second-order Kalman filtering model.*

**Figure 2:**
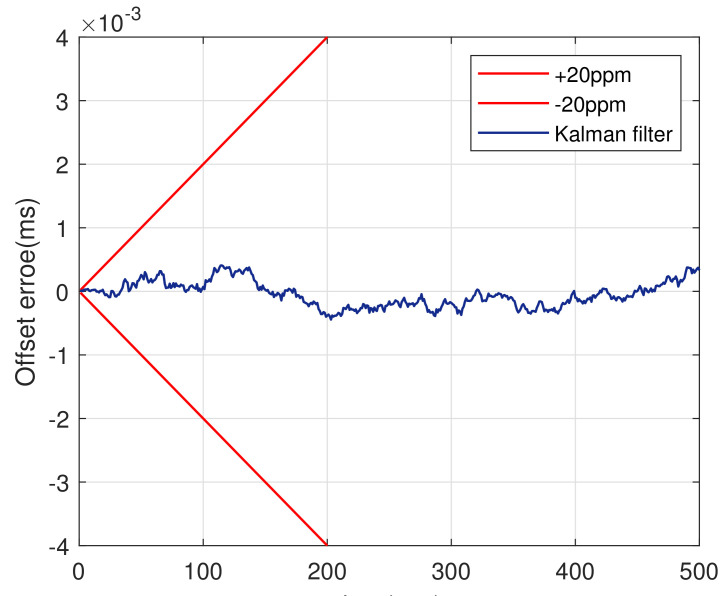
Illustration of clock drift.

Here, assuming a typical clock drift correspond to our mathematical model (e.g., ±20 ppm at a constant temperature) as the input gets. It is known from [34] that the measurement without observation noise of Kalman filter is τ×(±20 ppm). The Kalman filter can be run by substituting it into the measurement equation. The clock offset error fluctuates around 0 after Kalman filtering, as shown in Figure 2.

## 4. Adaptive Robust Synchronization Algorithm

It is well known that Kalman filtering is widely used in variable estimation because of its high accuracy and low complexity. In this paper, the discrete Kalman filtering is used to estimate and predict clock offset, which is feasible and beneficial. As shown in the Figure 1, during the time stamp transmission, the node B converts the actual interval $t_{n+1}-t_{n}$ into the interval according to its local clock $C_{B}\left(t_{n+1}\right)-C_{B}\left(t_{n}\right)$. However, the Kalman filtering may be unstable, and the measurement value may be abnormal due to the possible loss of measurement. Therefore, an adaptive robust synchronization(ARS) framework is proposed, which makes it reasonable for the reference node to adjust the sampling interval τ to send timestamp information, as needed.

### 4.1. Single-Hop Networks Scenario

The measured clock offset can be directly used to synchronize the slave clock. However, in practical applications, due to the influence of uncertainties, such as measurement noise and transmission delay, the measured values need to be filtered. If too many observation value are lost, the Kalman filtering process will be unstable. If the clock parameter estimation is not accurate, the clock of sensor nodes cannot be synchronized accurately. When considering the loss of observation, it is necessary to adjust the parameter of the Kalman filtering model. By analyzing the iterative process of error covariance, the relationship between the convergence of packet reception rate λ and covariance matrix expectation E[P(k)] is discussed [13].

Based on the above clock synchronization model, the Kalman filtering formula is derived as [35]:

(15) $$\hat{x}\left[k+1|k\right]=A\hat{x}\left[k\right]$$

(16) $$P\left[k+1|k\right]=AP\left[k\right]A^{T}+Q$$

(17) $$G\left[k+1\right]=P\left[k+1|k\right]H^{T}{\left(HP\left[k\right]H^{T}+R\right)}^{-1}$$

(18) $$\hat{x}\left[k+1\right]=\hat{x}\left[k+1|k\right]+\varepsilon \left[k+1\right]G\left[k+1\right]\left(z\left[k+1\right]-H\hat{x}\left[k+1|k\right]\right)$$

(19) P[k+1]=P[k+1|k]−ε[k+1]G[k+1]HP[k+1|k]

According to the above Kalman filtering formulas and let P[k|k−1]=P[k], the recursive equation of the prior error matrix can be obtained as:

(20) $$P\left[k+1\right]=AP\left[k\right]A^{T}+Q-\varepsilon \left[k\right]AP\left[k\right]H^{T}{\left(HP\left[k\right]H^{T}+R\right)}^{-1}HP\left[k\right]A^{T}$$

Next, the convergence properties of $\lim_{k\to \infty }E\left[P\left(k\right)\right]$ in the statistical sense are discussed.

(21) $$E\left[P\left(k+1\right)\right]=AE\left[P\left(k\right)\right]A^{T}+Q-\lambda AE\left[P\left(k\right)\right]H^{T}{\left(HE\left[P\left(k\right)\right]H^{T}+R\right)}^{-1}HE\left[P\left(k\right)\right]A^{T}$$

The following lemma is proposed in ref. [13] and ref. [35]

**Lemma** **1.** *If $\left(A,\sqrt{Q}\right)$ is controllable, (A,H) is measurable, and $\lambda >\lambda_{c}$, then when $\forall E[P_{0}]\geq 0$, there are 0<L(k)≤E[P(k)]≤U(k), $\lim_{k\to \infty }L\left(k\right)=\bar{L}$, $\lim_{k\to \infty }U\left(k\right)=\bar{U}$, where $\bar{L}$ is the solution of equation $\bar{L}=u_{\lambda }\left(\bar{L}\right)$, $\bar{U}$ is the solution of equation $\bar{U}=g_{\lambda }\left(\bar{U}\right)$, and $\lambda_{c}$ is packet reception rate, $\lambda_{c}\in \left[0,1\right]$, $u_{\lambda }\left(X\right)=\left(1-\lambda \right)AXA^{T}+Q$, $g_{\lambda }\left(\cdot \right)$ is the modified algebraic Riccati equation:*

(22) $$g_{\lambda }\left(X\right)=AXA^{T}+Q-\lambda AXH^{T}{\left(HXH^{T}+R\right)}^{-1}HXA^{T}$$

In wireless networks, energy issues are an important challenge for clock synchronization. The sending and receiving of synchronous timing messages are the main source of energy consumption. On the other hand, in many applications, synchronization offset within a specified range can be allowed, and different applications need different clock synchronization accuracy [36]. In our periodic synchronization strategy, the sampling period determines the performance of clock synchronization. Our challenge is to set a reasonable sampling period while ensuring the required accuracy.

The Kalman filter is unstable in IoT, according to the loss of data packets. The upper and lower bounds of error covariance are given by the above formulas. Additionally, the error covariance matrix at time k+1 can be predicted through the Kalman filtering at time *k*. From Equation (16), the variance of one-step prediction clock offset is obtained by the one-step prediction covariance matrix.

Therefore, the adaptive on-demand clock synchronization method is need to be adopted in synchronization process in order to ensure the clock accuracy. If

(23) $$M_{1,1}\left(k+1\right)\leq {\left(\frac{\gamma }{\sqrt{2}erf^{-1}\left(p\right)}\right)}^{2}$$

where $M_{1,1}\left(k+1\right)$ represents the elements of the first row and first column of one-step prediction error variance matrix, which is, clock offset. This paper assume that the clock offset between synchronization node and reference node is less than the preset value γ[37], and its probability is at least *p*. The relationship between γ and *p* satisfies p=Pr(|θ(n)|<γ), $erf^{-1}\left(\cdots \right)$ is an inverse Gaussian error function. If $M_{1,1}\left(k+1\right)$ does not satisfy the above inequality, the algorithm will adaptively adjust the sampling period τ.

Next, the adjustment of the sampling period τ has to be discussed. For 0<L(k)≤E[P(k)]≤U(k), where $\lim_{k\to \infty }U\left(k\right)=\bar{U}$, and by the equation $\bar{U}=g_{\lambda }\left(\bar{U}\right)$, $\bar{U}$ is a semi-positive definite matrix, as is known that ${\bar{U}}_{1,1}$ is a function about the sampling period τ, denoted ${\bar{U}}_{1,1}=f\left(\tau \right)$, where ${\bar{U}}_{1,1}$ represents the elements of the first row and the first column of the matrix $\bar{U}$, which is, the upper bound of the variance of clock offset error. If $M_{1,1}\leq {\left(\frac{\gamma }{\sqrt{2}erf^{-1}\left(p\right)}\right)}^{2}$, just need ${\bar{U}}_{1,1}\left(k+1\right)\leq {\left(\frac{\gamma }{\sqrt{2}erf^{-1}\left(p\right)}\right)}^{2}$, it is easy to get the maximum value of sampling interval τ by the following equation:

(24) $${\bar{U}}_{1,1}={\left(\frac{\gamma }{\sqrt{2}erf^{-1}\left(p\right)}\right)}^{2}$$

Because ${\bar{U}}_{1,1}$ is a function about the sampling period τ, and ${\bar{U}}_{1,1}=f\left(\tau \right)$. For function f(τ), $f({\bar{U}}_{1,1},\tau )=0$ is obtained from $\bar{U}=g_{\lambda }\left(\bar{U}\right)$, ie.

(25) $$\bar{U}=A\bar{U}A^{T}+Q-\lambda A\bar{U}H^{T}{\left(H\bar{U}H^{T}+R\right)}^{-1}H\bar{U}A^{T}$$

For first-order Kalman filter, the above formula can be written as:

(26) $$\begin{array}{cc} \left(\begin{array}{cc} {\bar{U}}_{1,1} & {\bar{U}}_{1,2}\\ {\bar{U}}_{2,1} & {\bar{U}}_{2,2} \end{array}\right)= & \left(\begin{array}{cc} 1 & \tau \\ 0 & 1 \end{array}\right)\left(\begin{array}{cc} {\bar{U}}_{1,1} & {\bar{U}}_{1,2}\\ {\bar{U}}_{2,1} & {\bar{U}}_{2,2} \end{array}\right)\left(\begin{array}{cc} 1 & 0\\ \tau & 1 \end{array}\right)+\left(\begin{array}{cc} \sigma_{\xi }^{2} & 0\\ 0 & \sigma_{\mu }^{2} \end{array}\right)-\lambda \left(\begin{array}{cc} 1 & \tau \\ 0 & 1 \end{array}\right)\left(\begin{array}{cc} {\bar{U}}_{1,1} & {\bar{U}}_{1,2}\\ {\bar{U}}_{2,1} & {\bar{U}}_{2,2} \end{array}\right)\\ \; & \left(\begin{array}{c} 1\\ 0 \end{array}\right){\left(\left(\begin{array}{cc} 1 & 0 \end{array}\right)\left(\begin{array}{cc} {\bar{U}}_{1,1} & {\bar{U}}_{1,2}\\ {\bar{U}}_{2,1} & {\bar{U}}_{2,2} \end{array}\right)\left(\begin{array}{c} 1\\ 0 \end{array}\right)+\sigma_{v}^{2}\right)}^{-1}\left(\begin{array}{cc} 1 & 0 \end{array}\right)\left(\begin{array}{cc} {\bar{U}}_{1,1} & {\bar{U}}_{1,2}\\ {\bar{U}}_{2,1} & {\bar{U}}_{2,2} \end{array}\right)\left(\begin{array}{cc} 1 & 0\\ \tau & 1 \end{array}\right) \end{array}$$

Simplify to get the system of equations:

(27) $$\left\{\begin{array}{c} \sigma_{\mu }^{2}-\frac{\lambda }{{\bar{U}}_{1,1}+\sigma_{v}^{2}}{\bar{U}}_{1,2}=0\\ \tau {\bar{U}}_{2,2}-\frac{\lambda }{{\bar{U}}_{1,1}+\sigma_{v}^{2}}\left({\bar{U}}_{1,1}{\bar{U}}_{1,2}-\tau {\bar{U}}_{1,2}\right)=0\\ 2\tau {\bar{U}}_{1,2}+\tau^{2}{\bar{U}}_{2,2}+\sigma_{\xi }^{2}-\frac{\lambda }{{\bar{U}}_{1,1}+\sigma_{v}^{2}}{\left({\bar{U}}_{1,1}+\tau {\bar{U}}_{1,2}\right)}^{2}=0 \end{array}\right.$$

By solving the system of equations, the following equation is obtained:

(28) $$\tau =\frac{\left[\lambda {\bar{U}}_{1,1}^{2}-\sigma_{\xi }^{2}\left({\bar{U}}_{1,1}+\sigma_{v}^{2}\right)\right]\sqrt{\lambda }}{\left[\left(2-\lambda \right){\bar{U}}_{1,1}+2\sigma_{v}^{2}\right]\sqrt{\sigma_{\mu }^{2}\left({\bar{U}}_{1,1}+\sigma_{v}^{2}\right)}}$$

We denote it as $f({\bar{U}}_{1,1},\tau )=0$. From the above equation, the maximum sampling period τ is obtained. Similarly, the maximum sampling period of the second-order Kalman filter can be obtained [38].

The solution of above equation can be used reasonably in order to ensure the synchronization accuracy. The solution is obviously more lenient and approximates the maximum sampling period $\tau_{max}$.

On the other hand, due to the existence of various random noises in the calculation and measurement of clock offset and skew, outliers [13,15,16] may exist in the filtered system state, which seriously reduces the reliability of data. However, the Kalman filtering algorithm is highly dependent on data, which is easily influenced by outliers and affect the filter accuracy. It is known that the fading memory Kalman filtering algorithm is trying to increase the effect of new measurement data and relatively reduce the impact of premature measurement data, which is, to strengthen the effect of new measurement data *z* on the Kalman filtering gain matrix *K* [28]. For the existence of outliers, a robust fading memory Kalman filtering algorithm is adopted to determine whether the new measurement data is outliers. If there is an outlier in the measurement data, the impact of the new measurement data will be reduced, and the influence of the previous measurement data is relatively strengthened. For systems (13) and (14), the robust Kalman filtering is:

(29) $$\hat{x}\left[k+1\right]=A\hat{x}\left[k\right]+\varepsilon \left[k+1\right]G\left[k+1\right]\left(z\left[k+1\right]-HA\hat{x}\left[k\right]\right)$$

(30) $$P\left[k+1|k\right]=\left(I-e\left[k+1\right]c\right)AP\left[k\right]A^{T}+Q$$

(31) $$G\left[k+1\right]=P\left[k+1|k\right]H^{T}{\left(HP\left[k\right]H^{T}+R\right)}^{-1}$$

(32) P[k+1]=P[k+1|k]−ε[k+1]G[k+1]HP[k+1|k]

In this paper, $e_{k}$ is defined to describe outlier events in the *k*th round of information exchange. When there are no outliers, $e_{k}=0$, error variance of one-step prediction does not change; when outliers appear, there is $e_{k}=1$. The gain matrix of Kalman filtering can be reduced by affecting the one-step prediction error variance. At this time, the gain effect of the new measurement data is reduced. Generally, the value of *c* is $e^{-l}$, where *l* is a constant that is greater than zero. For the problem of outliers, Layda’s is used criterion to determine whether the innovation is a multiple of the square root of clock offset error variance:

(33) $$o\left[k\right]=|z\left[k\right]-H\hat{x}\left[k+1|k\right]|>m\times \sqrt{HP\left[k\right]H^{T}}$$

Under normal circumstances, innovation is a Gaussian distribution with zero mean. We take the multiple m=3.

Because $P\left[k+1|k\right]=\left(1-e\left[k+1\right]c\right)AP\left[k\right]A^{T}+Q\leq AP\left[k\right]A^{T}+Q$, the upper bound of convergence of $\lim_{k\to \infty }E\left[P\left(k\right)\right]$ is still true, which is, 0<E[P(k)]≤U(k), then the adaptve robust on-demand clock synchronization algorithm with adaptively adjust sampling period is still available.

In the next section, we extend to the proposed clock synchronization algorithm in multi-hop networks.

### 4.2. Multi-Hop Networks Scenario

**Figure 3:**
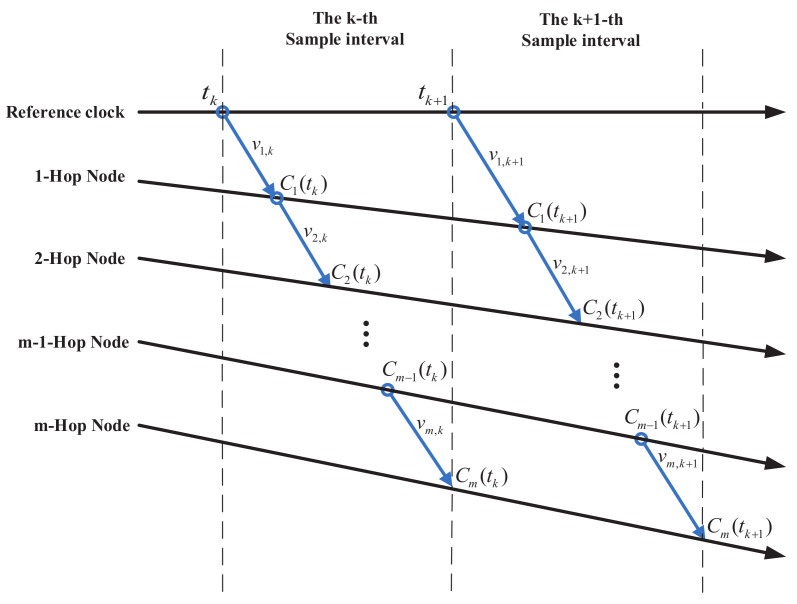
Multi-hop Clock Synchronization Process.

In the above analysis, the single-hop clock synchronization have been discussed. However, multi-hop clock synchronization is common in real life [39]. Multi-hop clock synchronization is even more challenging. The clock accuracy of multi-hop networks is closely related to the performance of each communication link. The proposed synchronization strategy can be extended to multi-hop network scenarios. Figure 3 shows the multi-hop clock synchronization process:

For the *m*-hop node, the acceptance rate of time-stamped data packets sent from the (m−1)-hop node is $\lambda_{m}$. Because each node forwards the time-stamped data packets, the *m*-hop packet reception rate is $\psi_{m}=\prod_{i=1}^{m}\lambda_{i}$. When the observation noise is $v_{k}$ at the *k*th sampling interval, the cumulative observation noise corresponding to the *m*-hop node and the reference time $t_{k}$ is described as $\phi \left(m,k\right)=\sum_{i=1}^{m}v_{i,k}$. Assuming that $v_{i,k}$ is independent, identically distributed and has Gaussian white noise with covariance $R_{v_{i,k}}$, then $R_{\phi_{m}}=\sum_{i=1}^{m}R_{v_{i,k}}$, where $R_{\phi_{m}}$ is the covariance relative to ϕ(m,k). It can be seen that it is only affected by two factors: the hop count *m* and the covariance $R_{v_{i,k}}$ of measurement noise in per hop.

Therefore, the Kalman filtering formula is derived, as follows:

(34) $$\hat{x}\left[k+1\right]=\hat{x}\left[k+1|k\right]+\varepsilon \left[k+1\right]G\left[k+1\right]\left(z\left[k+1\right]-H\hat{x}\left[k+1|k\right]\right)$$

(35) $$P\left[k+1|k\right]=\left(I-e\left[k+1\right]c\right)AP\left[k\right]A^{T}+Q$$

(36) $$G\left[k+1\right]=P\left[k+1|k\right]H^{T}{\left(HP\left[k\right]H^{T}+\sum_{i=1}^{m}R_{v_{i,k}}\right)}^{-1}$$

(37) P[k+1]=P[k+1|k]−ε[k+1]G[k+1]HP[k+1|k]

In a multi-hop network, the convergence property of $\lim_{k\to \infty }E\left[P_{k}\right]$ in a statistical sense.

(38) $$E\left[P_{k+1}\right]=AE\left[P_{k}\right]A^{T}+Q-\prod_{i=1}^{m}\lambda_{i}E\left[AP_{k}H^{T}{\left(HP_{k}H^{T}+\sum_{i=1}^{m}R_{v_{i,k}}\right)}^{-1}HP_{k}A^{T}\right]$$

**Corollary** **1.** *For multi-hop networks, if $\left(A,\sqrt{Q}\right)$ is controllable, (A,H) is measurable, and $\lambda >\lambda_{c}$, then when $\forall E[P_{0}]\geq 0$, there are 0<L(k)≤E[P(k)]≤U(k), $\lim_{k\to \infty }L\left(k\right)=\bar{L}$, and $\lim_{k\to \infty }U\left(k\right)=\bar{U}$, where $\bar{L}$ is the solution of equation $\bar{L}=\left(1-\prod_{i=1}^{m}\lambda_{i}\right)A\bar{L}A^{T}+Q$, $\bar{U}$ is the solution of equation $\bar{U}=h_{\lambda }\left(\bar{U}\right)$, and $\lambda_{c}$ is the critical package acceptance rate, $\lambda_{c}\in \left[0,1\right]$, $h_{\lambda }\left(\cdot \right)$ is the modified algebraic Riccati equationv:*

(39) $$h_{\lambda }\left(X\right)=AXA^{T}+Q-\prod_{i=1}^{m}\lambda_{i}AXH^{T}{\left(HXH^{T}+\sum_{i=1}^{m}R_{v_{i,k}}\right)}^{-1}HXA^{T}$$

If observation information (a few timestamps from its upstream nodes) is available, then each node in the multi-hop network can predict clock offset and estimate clock skew according to the method proposed for single-hop scheme. In addition, it is easy to see that, the greater distance from the reference node, the greater clock error. Therefore, the maximum sampling period in the synchronization strategy should be designed based on the error variance of the farthest node in order to ensure the synchronization accuracy of entire multi-hop network.

## 5. Simulations

In this section, the ARS algorithm is simulated in Matlab. We conduct the ARS scheme in the presence of packet loss to verify the performance of algorithm. By comparing with existing algorithms, the superiority of ARS algorithm is shown. The simulation consists of the following three parts: (1) for single-hop networks, verify the clock synchronization between the ARS scheme proposed with the first-order Kalman filter and the existing scheme. In addition, the relationship between sampling period τ and clock offset error was verified through simulation; (2) in the single-hop network, compare the clock synchronization between the ARS algorithm with the second-order Kalman filter and the existing scheme. To illustrate the adaptability of the algorithm, the sampling interval τ of each sampling period *k* is described; and, (3) for multi-hop networks, verify the clock synchronization between the ARS scheme proposed with the first-order Kalman filter and the existing scheme. Firstly, the measurement of synchronization error is given with the same packet loss probability. Subsequently, the performance comparison is conducted to illustrate the importance of ARS algorithm.

Note that the low-cost crystal oscillators in wireless sensor networks can be affected by as voltage, temperature, and humidity [6]. In the underground mine, it is a high temperature, high humidity environment, and the voltage is unstable. Therefore, the clock reading is not accurate, resulting in setting larger state noise and measurement noise according to the actual environment. Additionally, the state estimation results of the Kalman filter depend on the influence of measurement noise. Therefore, the accuracy of clock synchronization scheme in coal mines is lower than that of the general wireless sensor network. In addition, due to the complexity of underground mining environment, the wireless sensor network is facing the problem of packet loss, which is also considered in the simulation experiments.

### 5.1. First-Order Kalman Filtering for Single-Hop Networks

Tracking experiments are carried out based on the first-order Kalman filtering firstly in order to describe the performance of Kalman filter on clock tracking. In our experiments, the clock aging rate is ignored so that it is equal to 0. In this case, the covariance of state noise $\omega_{k}$ and measurement noise $v_{k}$ are set to $Q=\left(\begin{array}{cc} \sigma_{\xi }^{2} & 0\\ 0 & \sigma_{\mu }^{2} \end{array}\right)=\left(\begin{array}{cc} 10^{-10} & 0\\ 0 & 10^{-12} \end{array}\right)$, $R=\sigma_{v}^{2}=10^{-8}$, the packet acceptance rate λ=0.8, and the initial value of sampling period τ to 2 s. The offset variance of synchronization nodes are expected to be less than the preset value $\gamma =5\times 10^{-9}$, with a probability of at least p=0.996.

**Figure 4:**
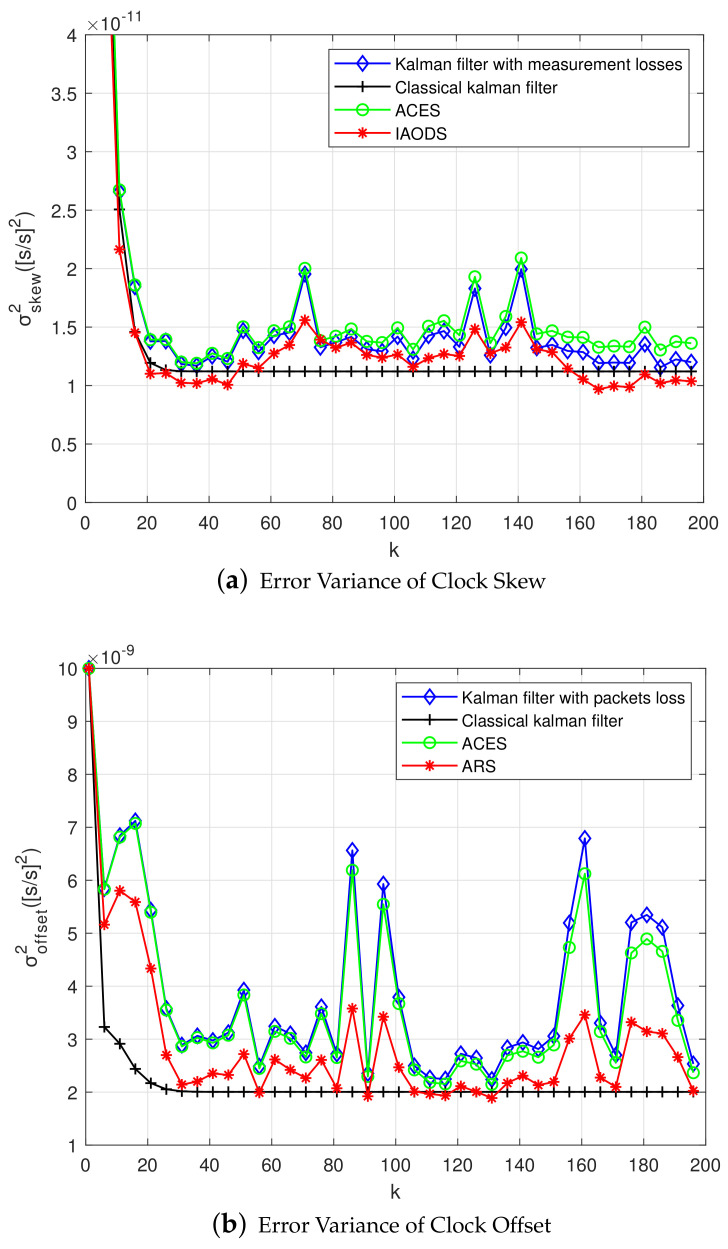
Error Variance of the First-order Kalman Filter in Single-hop Networks.

As shown in Figure 4a, the error variance of clock skew changes with the increase of sampling period. The true state of clock is tracking closely by Kalman filter, and the estimation of clock skew is close to the real value. At the same time, the estimation of clock skew is mainly affected by three factors: sampling interval, process noise, and observation noise. When the three factors are the same, the clock skew of the classical Kalman filter gradually stabilizes with the increase of the sampling period. While the Kalman filter with packet loss converges rapidly at first, but then fluctuates up and down, and it is close to the classical Kalman filter. In the case of packet loss, the ACES scheme does not reduce the error variance of clock skew, but increases. Finally, by verifying our proposed ARS scheme, the error variance of clock skew was significantly reduced. And around the skew convergence value of the classical Kalman filtering fluctuates.

Figure 4b shows the relationship between sampling period and the error variance of offset. The main purpose of Kalman filtering is to reduce the error variance of clock offset. It can be seen from the figure that in the ACES scheme, the error variance of clock offset is not significantly reduced compared to the Kalman filter with packet loss. However, the error variance of offset can be reduced by conducting the ARS scheme clearly. It is close to the case of classical Kalman filtering.

**Figure 5:**
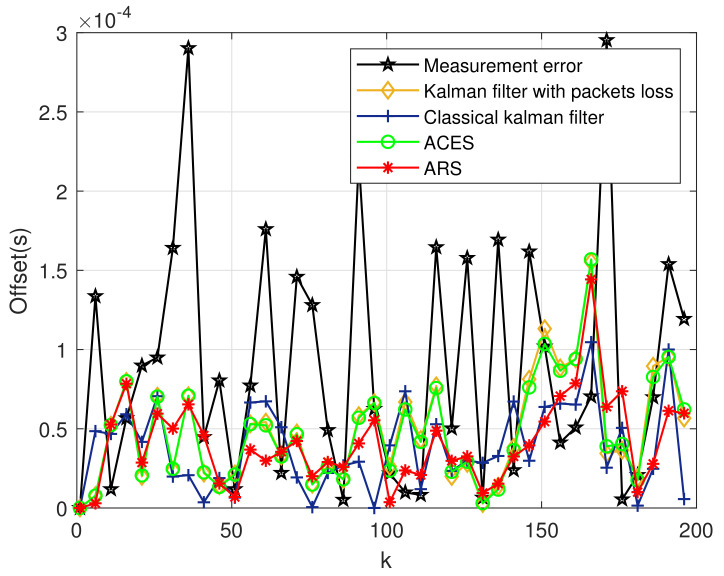
Clock Offset Error of the First-order Kalman Filter.

Next, Figure 5 shows the error of clock offset. It can be seen that the estimation error is significantly reduced compared with the measurement error, where the measurement error means x[n]−z[n] after Kalman filter processing. There is not much difference in the estimation error between several algorithms, but the improvement of clock offset error after the ARS algorithm is still shown. Meanwhile, we conclude that the stability of clock is strong in single-hop network, and the ARS algorithm with the first-order Kalman filter has little effect on clock offset.

**Figure 6:**
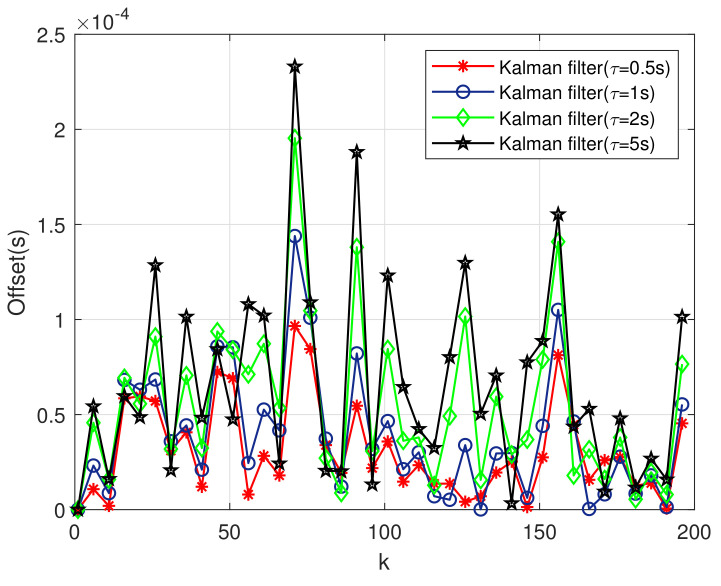
Clock Offset Error with Different Sampling Interval.

Take the first-order Kalman filter as an example, Figure 6 describes the dependence of the clock offset error on the sampling interval. Different sampling intervals have different clock offset errors, as shown in the figure. The longer the sampling interval is, the greater the clock offset error is. Therefore, we can reduce or increase the clock offset error by adjusting the sampling interval. At the same time, the increase of sampling interval can reduce the number of sampling, which is, the number of clock synchronization. It can reduce energy consumption. However, the clock offset error is increased. On the contrary, reducing the sampling interval increases the number of clock synchronization and increases the number of clock synchronization.

**Figure 7:**
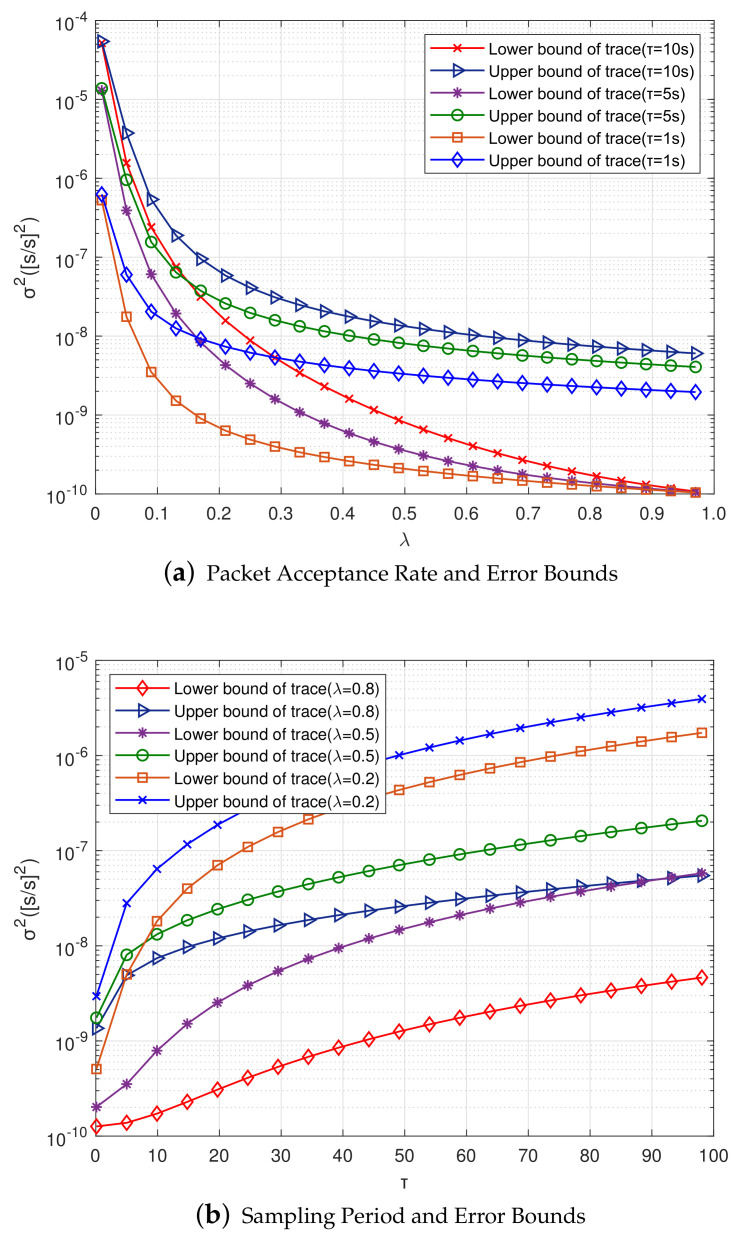
Relationships Between Different Factors and Error Bounds of the First-order Kalman Filter.

Figure 7a shows the relationship between packet reception rate λ and the upper and lower bounds of error covariance. After setting up different sampling periods, the traces of upper and lower bounds *U* and *L* gradually decrease as the packet reception rate λ gradually increases, indicating that $g_{\lambda }\left(U\right)$ and $u_{\lambda }\left(L\right)$ are monotonically decreasing functions about λ. In addition, the smaller the sampling period is, the smaller the convergence values of upper and lower bounds of covariance are. When the packet reception rate λ gradually transitioned from 0.01 to 1, TrU decreased by one order of magnitude, while TrL decreased by nearly two orders of magnitude, and finally, different sampling periods dropped to the same value. It shows that the size of packet reception rate affects clock synchronization accuracy, and the more clock information receives, the higher clock accuracy is.

In this paper, the upper bound $U_{k}$ is used as the condition of adaptive on-demand synchronization algorithm, and the threshold of offset error variance is set. At this time, it is necessary to analyze whether the current system synchronization accuracy meets the application requirements. If it is not satisfied, the clock synchronization is deemed unstable. As is shown in Figure 7b, the relationship between the upper and lower bounds of error variance and the sampling period, indicating that $g_{\lambda }\left(U\right)$ and $u_{\lambda }\left(L\right)$ are monotonically increasing functions about the sampling period. As the sampling period increases, the upper and lower bounds of covariance gradually increase. In addition, it is also found that according to different packet acceptance rates, the lower bound of the error variance is greater than the upper bound.

### 5.2. Second-Order Kalman Filtering for Single-Hop Networks

In this section, simulation experiments are conducted by using the second-order Kalman filtering in order to estimate clock skew. The same as the previous section, the Kalman filter is initialized as: $\sigma_{\rho }^{2}=10^{-14}$, $R=\sigma_{v}^{2}=10^{-8}$, the packet reception rate is λ=0.8, and the sampling period τ is initialized to 1 s.

**Figure 8:**
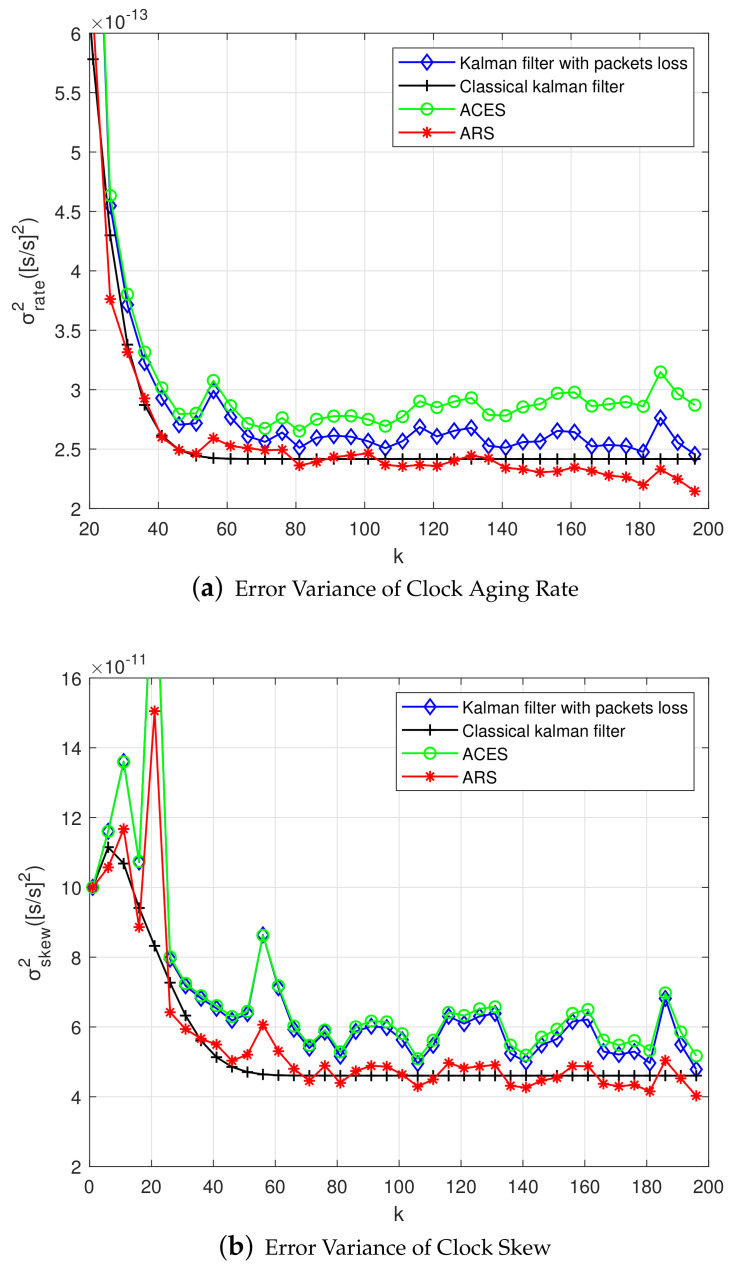
Error Variance of the Second-order Kalman Filter in Single-hop Networks.

Similar to the previous section, clock aging rate and skew are much lower than offset. Through the implementation of experiments, we find that the error variance of aging rate, skew, and offset are significantly reduced by the ARS algorithm. Figure 8a shows the error variance of clock aging rate using the second-order model. Figure 8b shows the error variance of skew. It is seen that, as the sampling period increases, the error variance decreases. Similar to the first-order Kalman filtering, the second-order Kalman filtering is unstable due to the loss of data packets, and this instability can be reduced by using the ARS algorithm.

**Figure 9:**
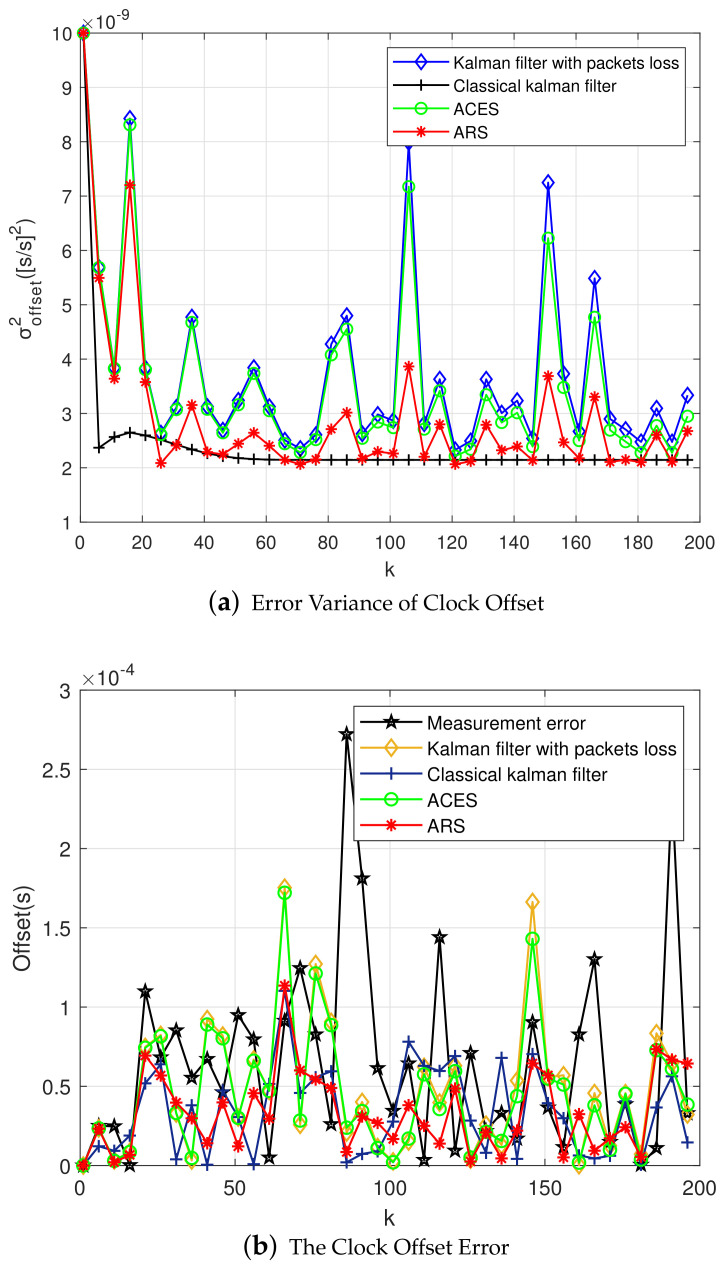
Clock Offset Error and Variance of the Second-order Kalman Filter in Single-hop Networks.

In Figure 9a, the error variance of clock offset is shown. It is illustrated that the error variance of offset is more unstable when compared with the first-order Kalman filtering. In addition, at some time, the error variance of clock offset is significantly larger than that of other times, and it seems that there is an outlier at this time. After processing by the ARS algorithm, the error variance of offset reduces below the required clock accuracy later, and the sudden increase of error variance when the outliers of the measured values occur is reduced. When compared with the first-order Kalman filter [38], clock offset error of the second-order Kalman filter is more obvious after being processed by the ARS algorithm, as shown in the second-order Kalman filter clock offset error that is shown in Figure 9b.

**Figure 10:**
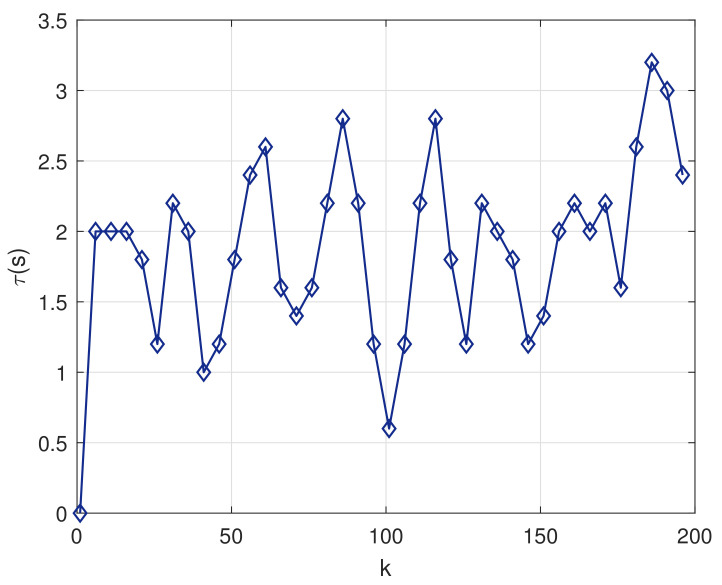
Variation of Sampling Interval τ Over Time.

Taking the second-order Kalman filter as an example, Figure 10 shows the change of sampling period when the Kalman filter is running for clock synchronization. The ARS algorithm can adjust the sampling interval adaptively according to the required clock accuracy. The sampling interval τ is obtained by Equation (28). It can be known that the factors influencing the size of sampling interval is determined by offset error covariance, packet loss rate, state noise covariance, and observation noise covariance. The longer the sampling interval, the smaller the energy consumption. It can not only meet the accuracy requirements, but also save energy consumption. It is very practical for coal mine underground environment.

**Figure 11:**
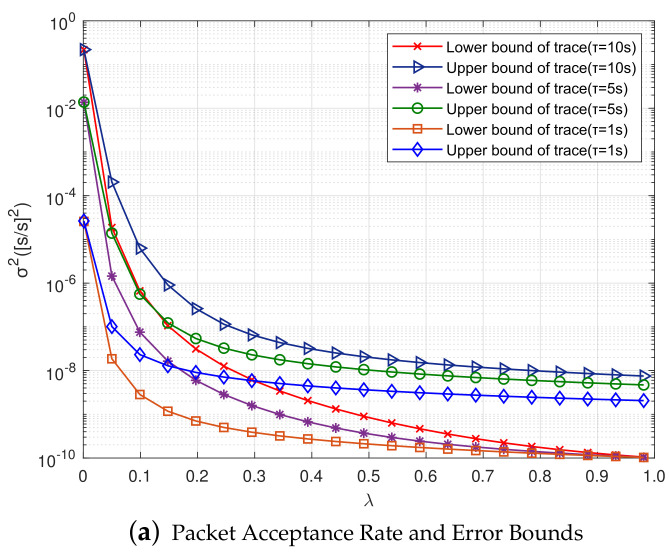

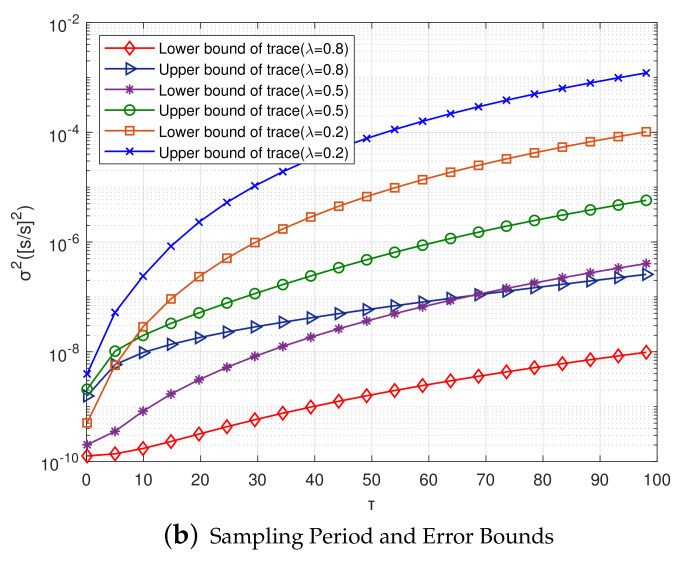
Relationships Between Different Factors and Error Bounds of the Second-order Kalman Filter.

Figure 11a shows the relationship between the second-order Kalman filter packet reception rate λ and the upper and lower bounds of error. When compared with the first-order Kalman filter, with the increase of packet reception rate, the traces of the upper and lower bounds *U* and *L* also gradually decrease, but the reduction speed is faster. The second-order Kalman filter also uses the upper bound $U_{k}$ as a condition for on-demand synchronization, and sets the threshold of the clock error variance. At this time, it is necessary to analyze whether the current system synchronization accuracy meets the application requirements. If it does not, the clock synchronization is deemed to be unstable.

The relationship between the upper and lower bounds of covariance and sampling period, as shown in Figure 11b. With a gradual increase of sampling period, the covariance also gradually increases, and the speed of the second-order Kalman filter increases faster than that of the first-order Kalman filter.

### 5.3. First-Order Kalman Filtering for Multi-Hop Networks

In this section, first-order Kalman filters under multi-hop nodes is discussed. Firstly, the Kalman filter should also be initialized: set the covariances of state noise $\omega_{k}$ and measurement noise $v_{i,k}$ to $Q=\left(\begin{array}{cc} \sigma_{\xi }^{2} & 0\\ 0 & \sigma_{\mu }^{2} \end{array}\right)=\left(\begin{array}{cc} 10^{-10} & 0\\ 0 & 10^{-12} \end{array}\right)$, $R_{\phi_{m}}=\sum_{i=1}^{m}\sigma_{v_{i,k}}^{2}=5\times 10^{-8}$, the packet acceptance rate $\psi_{m}=\prod_{i=1}^{m}\lambda_{i}=0.32$, and the hop count is five hops.

**Figure 12:**
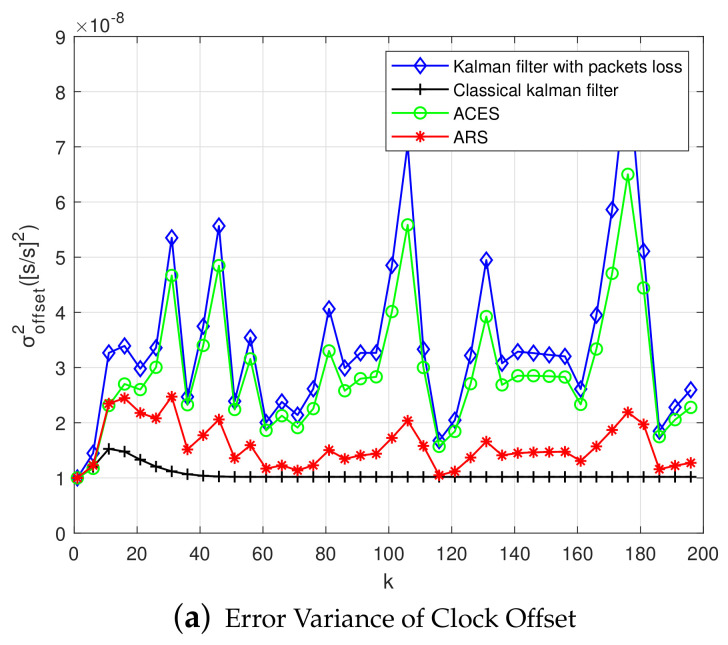

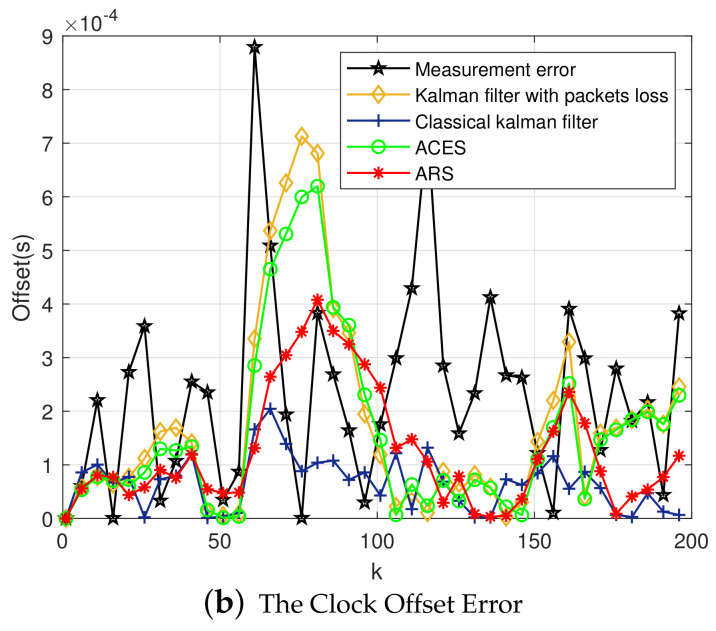
Clock Offset Error and Variance of the First-order Kalman Filter in Multi-hop Networks.

The clock synchronization of multi-hop nodes is closely related to the performance of each communication link. A layer-by-layer data exchange strategy is used to exchange information between the synchronization node and the reference node [37]. Due to the low packet reception rate and large measurement noise in multi-hop nodes, the error variance of clock offset is much larger than that of single-hop synchronization, as shown in Figure 12a. This paper adopts the adaptive robust synchronization algorithm, adjust the sampling period according to the required clock accuracy, and obviously reduce the error variance of offset. The clock offset error is shown in Figure 12b, and the offset error has also been greatly reduced. There is a big gap when compared with the ARS algorithm proposed by other existing solutions.

In the case of possible packet loss, the mean, maximum, and minimum values of clock synchronization error of the ARS algorithm are smaller than the Kalman filter with packet loss and the ACES algorithm, which is close to the classic Kalman filter algorithm, its fluctuation range is [42.36, 176.86], as can be seen from Table 2. The ARS algorithm can well reduce the impact of packet loss on synchronization accuracy and ensure the stability of operation.

Where KF stands for Kalman filter, case 1, case 2, and case 3 represent first-order Kalman Filter in single-hop networks, second-order Kalman filter in single-hop networks, and first-order Kalman filter in multi-hop networks, respectively.

In short, we come to the following conclusions. Firstly, in the single-hop network, the ARS algorithm improves the clock accuracy of the second-order Kalman filter better than that of the first-order Kalman filter. Secondly, in multi-hop networks, the reduction of clock offset error by the ARS algorithm is more obvious than in single-hop networks.

## 6. Conclusions

For resource-constrained IoT in underground mines, adaptive clock synchronization is still a challenge. In this paper, different Kalman filtering models and various experimental clock synchronization schemes are studied. Based on the observation model under incomplete measurement, the statistical error quantitative analysis of the variance boundary is performed on the synchronization error covariance in the case of packet loss. The ARS algorithm is designed to adaptively adjust the sampling period. Additionally, for the case where there are outliers in the measurement process, the ARS scheme can reduce the impact of outliers. This article starts from small-scale single-hop nodes and extends to multi-hop networks. For multi-hop node wireless sensor networks, achieving better robustness clock synchronization for unreliable IoT in underground mines. The simulation results show that the accuracy of ARS algorithm is improved by 7.85% when compared with previous studies in single-hop networks and improves the accuracy by 12.56% in multi-hop networks. The method in this paper has strong theoretical research and practical application value.

The future work will focus on the real environment of the mine, clock synchronization. Different sensor node devices have different internal clocks. Therefore, the deployment of sensor nodes that are suitable for underground mine needs to be solved. Another important direction of further investigation is how to improve the accuracy of the clock in the underground mines.

## Figures and Tables

**Table 1:** Symbols and functions description.

Symbols	Description	Symbols	Description
C(t)	ideal time	θ[n]	clock offset
α[n]	clock skew	β[n]	clock aging rate
ξ[n]	offset state noise	μ[n]	skew state noise
ρ[n]	aging rate state noise	x[n]	state vector
*A*	state transition matrix	ω[n]	state noise
z[n]	observation vector	*H*	observation matrix
*Q*	state noise covariance	*R*	observation noise covariance
ε[k]	random variable	$u_{\lambda }\left(X\right)$	convergent lower bound function
$g_{\lambda }\left(X\right)$	convergent upper bound function	γ	offset covariance threshold
e(k)	random variable	o[k]	innovation
$\psi_{m}$	reception rate	ϕ(m,k)	observation noise

**Table 2:** Comparison of clock error statistics results of different algorithms (μs).

Algorithm	Case 1			Case 2			Case 3		
	Mean	Max	Min	Mean	Max	Min	Mean	Max	Min
KF with Packets Loss	56.80	84.13	45.48	60.82	90.41	46.92	170.42	252.37	122.38
Classical KF	44.82	46.58	44.77	46.72	50.95	46.31	101.70	115.36	100.96
ACES	55.48	81.32	44.42	56.93	81.84	44.45	159.16	226.39	116.27
ARS	48.67	58.81	42.36	52.47	67.38	44.32	117.84	176.86	93.18
